# Designing Religiously Informed and Culturally Acceptable Tobacco Cessation Interventions for UK-Based Muslims

**DOI:** 10.1093/ntr/ntaf178

**Published:** 2025-08-28

**Authors:** Felix Naughton, Sylvia Barnes, Carole Gardener, Caitlin Notley, Rachna Begh, Nicola Lindson, Salman Waqar, Chloë Siegele-Brown, Aimie Hope

**Affiliations:** School of Health Sciences, University of East Anglia, Norwich NR4 7UL, UK; School of Health Sciences, University of East Anglia, Norwich NR4 7UL, UK; School of Health Sciences, University of East Anglia, Norwich NR4 7UL, UK; Medical School, University of East Anglia, Norwich NR4 7UL, UK; Nuffield Department of Primary Care Health Sciences, University of Oxford, Oxford OX2 6GG, UK; Nuffield Department of Primary Care Health Sciences, University of Oxford, Oxford OX2 6GG, UK; Department of Primary Care and Public Health, Imperial College London, London, UK; Faculty of Medicine, University of Southampton, Southampton, UK; School of Health Sciences, University of East Anglia, Norwich NR4 7UL, UK; Tyndall Centre, University of East Anglia, Norwich, UK

## Abstract

**Introduction:**

Globally, tobacco use rates in Muslim communities, particularly among men, are significantly higher than in non-Muslim communities. In the United Kingdom, there are also low rates of help-seeking among British Muslims who use tobacco. Ramadan could be a “window of opportunity” to support tobacco use behavior change but we lack the voice of British Muslim communities on culturally tailored cessation support. We undertook a public and patient involvement and engagement (PPIE) project to gain views from representatives of these communities.

**Aims and Methods:**

Discussions with 15 PPIE representatives from, or who worked with, a variety of British Muslim communities identified through gatekeepers, social media, and snowballing approaches. Key points and views from PPIE discussions were summarized into broad themes.

**Results:**

Opportunities and challenges with culturally adapting tobacco cessation support to Ramadan were raised. “Light touch” positive religious messaging connected to tobacco cessation was recommended, and overemphasizing religion in messaging content to be avoided. Quitting during Ramadan was felt challenging due to fasting, precluding the use of nicotine replacement products or medication, and reinforcing tobacco use as part of fast-breaking routines. Instead, PPIE representatives suggested quitting in advance of Ramadan or promoting cessation afterward by capitalizing on tobacco reduction achieved during Ramadan. There was support for digital cessation approaches, but it was felt many in their communities would prefer traditional approaches, including interpersonal support and messaging through influential community members.

**Conclusions:**

“Light touch” culturally tailored tobacco cessation support before or after Ramadan was felt more promising than supporting cessation initiation during Ramadan.

**Implications:**

Members of British Muslim communities identified challenges with using Ramadan as a “window of opportunity” for tobacco behavior change and favored quitting ahead of time *for* Ramadan or capitalizing on tobacco behavior change achieved during Ramadan to promote a quit attempt afterward. Taking a “light touch” approach with religiously tailored messaging could help engage Muslim people who smoke in cessation support. However, avoiding any strong or negatively framed tobacco-related messages linked to religious phrases or imagery is important. This work reinforces the importance of engaging with communities when considering culturally adapting interventions to prevent misdirected adaptions.

## Introduction

Globally, smoking rates are high in Muslim communities, particularly among men and in countries where Muslims are a minority.^1^ In England in 2023, 13% of men aged 18 and over smoked cigarettes versus 16% of Muslim men—the highest rate across all religious groups.^2^ Furthermore, some specific groups within Muslim communities have much higher tobacco use rates. For example, smoking rates among male Somalis are reportedly as high as 33% in the United Kingdom^3^ and 44% in the United States.^4^ British Muslims are a relatively young and ethnically diverse set of communities with disproportionate levels of socioeconomic deprivation,^5^ which may contribute to the higher than average tobacco use rates.

Smoking rates among adult female Muslims in the United Kingdom are comparatively low (4%).^2^ However, these rates are thought to be underestimated due to underreporting.^6^ Studies show Muslim women who smoke are likely to engage in secretive smoking practices due to fear of social judgment, in part due to culturally informed differences in gender identities.^7^ The true tobacco use rate among UK Muslim women is, therefore, largely unknown.

There is also diversity in how people from many Muslim communities use tobacco, including through water pipes and chewing tobacco. Such products are often not accommodated in smoking rate statistics or tobacco cessation interventions and are poorly regulated, particularly in countries where these are minority use products.^8^

Muslims who smoke are also less likely to succeed in quitting smoking than Christian or nonreligious populations in England.^9^ Most quit attempts by Muslims are unassisted, with low rates of help-seeking from health services. A London-based study found family and friends were the most commonly accessed source of help for quitting smoking by Muslims, with only a small minority reporting awareness of specialist stop smoking services.^10^ This may reflect the multiple barriers to accessing evidence-based tobacco cessation treatments experienced by many UK Muslims. A study of UK-based Pakistani and Bangladeshi people who smoked, their families and their healthcare professionals, identified barriers to making interventions culturally appropriate, including: language, religion, culture, time pressures, and a deficit of resources.^11^ Despite repeated calls for developing and increasing access to culturally tailored tobacco cessation support^6^^,^^12^ and evidence that this increases the effectiveness of cessation support,^13^ there has been little progress. One potential opportunity proposed is to tailor cessation support around Ramadan.

Ramadan is the ninth month in the Islamic calendar where Muslims fast during the day and engage in prayer and reflection. Ramadan could be a “window of opportunity” for tobacco behavior change,^12^^,^^14^ through high compliance with abstaining from tobacco during daylight hours combined with changes in environmental cues and social norms. Research from Malaysia shows that 98% of Muslims who smoke report reducing their smoking rate during Ramadan and 97% feel that quitting during Ramadan would be easier than outside of Ramadan.^15^ Similarly, a study of London-based Muslims showed comparable views about Ramadan being a suitable time to quit tobacco use.^10^ Preliminary research in the United States, investigating digital cessation support delivered to Muslims who smoked during Ramadan, has also shown promise,^14^ as has a booklet-based intervention in Malaysia for extending smoking-behavior change beyond Ramadan.^16^

However, questions remain over whether Ramadan is a suitable time to promote tobacco cessation. Abstaining from food can increase tobacco urges, and oral medications, including nicotine replacement therapies, are precluded during daylight hours.^12^^,^^17^ There is also concern that quitting motivation may not remain beyond Ramadan, once the month’s “strong spiritual drive” has subsided.^18^

We lack the voice of British Muslim communities on how best to deliver culturally tailored cessation support. As a first step in addressing this gap, we undertook a public and patient involvement and engagement (PPIE) project focused on culturally tailored cessation support for UK Muslims who use tobacco. We focused on understanding people’s experiences of tobacco use during Ramadan and their views on potential design features of tobacco cessation support tailored around Ramadan.

## Methods

This public involvement project identified people from, or who worked with, a variety of Muslim communities in England. People were identified primarily through “gatekeepers”—people with strong links with different Muslim communities who were either a recognized community champion or had existing community roles linked to health promotion and/or outreach. We shared promotional information on social media, on the University of East Anglia’s message boards, and directly contacted relevant religious organizations and community groups across the country by email to identify PPIE representatives. Organizations and groups included multiple mosques in the East of England region, the British Islamic Medical Association, and the Born in Bradford PPIE panel. As a secondary identification method, we used snowballing techniques.

PPIE discussions were held in-person, most often in mosques, or online. In some cases, to maximize inclusivity, other PPIE representatives provided translation where English was not spoken fluently by representatives. Meetings were guided by a semi-structured guide. This included the following topics: experiences of tobacco use and quitting use during Ramadan; supporting people in quitting tobacco; tobacco cessation treatment preferences, particularly digital approaches, and what research is needed to investigate these; how the month of Ramadan could be used to tailor tobacco cessation support; and how best to engage different Muslim community groups. Extensive field notes were taken during the meetings with key points and views from these discussions summarized into broad themes. A formal thematic analysis was not undertaken given the nature of the project. As is typical with UK PPIE projects, and according to guidelines from the UK Health Research Authority, formal ethical approval was not required, although all activities were ethically conducted and informed by the UK Standards for Public Involvement.^19^

## Results

Fifteen PPIE representatives, including tobacco users, ex-users, and non-users, participated in discussions across 10 meetings. There were 10 females and 5 males, who represented a diverse range of England-based Muslim communities, with South Asian, African, and Middle Eastern ethnicities, and mainly resided in Northern England, Eastern England, or London. Two representatives had experience working with these communities but were not from a Muslim community themselves.

Key viewpoints from discussions relating to informing future efforts to support tobacco cessation in these underserved communities were split into three broad themes below.

### Supporting People within Muslim Communities to Quit Tobacco

PPIE representatives supported the notion of enabling people to quit or prepare to quit before or during Ramadan. However, it was highlighted that planning a quit attempt during Ramadan could be difficult because participants are under a lot of pressure due to food cravings. Furthermore, it was felt that tobacco users really look forward to having a cigarette or tobacco when they break the fast, adding to the challenge. However, some Muslims prepare for Ramadan by voluntarily fasting before it starts, and it was felt this could be replicated with tobacco cessation.

There was support for a potential role of a technology-orientated approach for delivering tobacco cessation support. However, it was noted that interventions, such as a smartphone apps, may not work for everyone. Older populations may be more likely to respond to traditional approaches, such as messaging at mosques or in-person support. It was also suggested that text messages may be preferable to an app as there would be no need to log-in or download anything to receive them. Talking therapies were highlighted as a potential means of addressing underlying anxiety, depression, and isolation, which were considered to be drivers of tobacco use. It was suggested that these should be respectful of people’s decisions to use tobacco but also help to address underlying tobacco-related issues. A further clear message was the importance of paying attention to the diversity of the community when developing support.

### Religious-Oriented Stop Tobacco Messaging

There was caution about over-emphasizing religion in tobacco cessation content, with a “light touch” approach recommended. It was felt that religious messaging, especially when framed negatively, could be demotivating and risk causing offense. Health messaging was deemed more appropriate, although it was recognized that people vary in how receptive they are to this. Religiously inspired phrases and evocative imagery were suggested as potentially useful for communications, as well as targeted messaging such as:

- Any focus on Ramadan as being about doing good deeds, eg, donating money saved from not using tobacco to the mosque;
- The quality of worship being better without distraction due to cravings;
- The potential value of being “ready” to quit over Ramadan;
- The impact of second-hand tobacco smoke on friends and family; and
- Encouragement to engage in prayers and Ramadan practices to distract from tobacco use.

The look, wording, and design of support was deemed important, and care was advised around the use of images promoting tobacco cessation support to ensure these would be unobtrusive. For example, some felt that people might take offense at pictures of ashtrays on materials including the word “Ramadan.” Views were that the wording should steer clear of suggesting that Muslims have a problem or are being targeted or vilified because of their tobacco use. A message such as: “Would you like help with quitting for Ramadan?” was deemed more acceptable.

Alternative times to Ramadan as an opportunity for tobacco cessation were also provided, including Hajj (the pilgrimage to Makkah), Muharram (the month starting the Islamic New Year), and the period between 1 year’s Ramadan and the next (ie, using each Ramadan as a “goal post”). Support tailored more generically around “40 days” was suggested because some Muslim communities have traditions reflecting this duration.

### Promoting Tobacco Cessation Support within Muslim Communities

PPIE representatives felt there was potential value in faith-based support and that this could take different forms. For example, scholars and doctors at local mosques could be influential in spreading health messages, often more so than Imams. One approach could be to engage with influential members of the communities who could promote support or messages related to tobacco cessation, particularly regarding the impact of tobacco on members of the Muslim community. Media could also play a role in this, eg, community radio stations and ethnic media channels. The Federation of Islamic Medical Associations and the British Islamic Medical Association, as examples of faith-based organizations of clinicians, could be useful spokespeople for future services. General Practitioners (GPs) were also cited as highly respected sources of information.

## Discussion

PPIE representatives of British Muslim communities highlighted opportunities as well as challenges with providing culturally adapted tobacco cessation support. Opportunities included using positive and religiously aligned messaging to highlight the benefits of quitting, potentially linked to Ramadan or other key Islamic-related events. A “light touch” approach was advocated if using religiously tailored messaging, with strong or negatively framed tobacco-related messages linked to religious phrases or imagery not considered appropriate. Spreading key messages and providing information for taking up cessation support via influential members and opinion leaders of Muslim communities was seen as effective for reaching tobacco users, though research highlights that care is required to ensure smoking-related health promotion programs are adapted to individual organizations.^20^ Digital approaches were seen as appropriate for some, but unlikely to appeal to all members of communities, who would likely prefer interpersonal interactions, including support that addresses wider needs, such as mental health-related factors.

While there was enthusiasm for Ramadan-linked tobacco cessation support, as found by others,^14^ several challenges were raised by PPIE representatives in starting a quit attempt *during* Ramadan. These included the additional challenge of managing food cravings during daylight hours, which can worsen tobacco cravings,^17^ without being able to use nicotine replacements, as this would be considered to invalidate a person’s fast. In addition, some tobacco users felt the first cigarette after breaking the fast would be particularly difficult to resist. PPIE representatives expressed that quitting in advance of and so *for* Ramadan was more suitable and more likely to be successful. This meant that when Ramadan started, they should be through the hardest part of their attempt. Alternatively, support could be developed to start immediately after Ramadan ends, to capitalize on tobacco reduction achieved during the holy month.

Key limitations included the challenges of capturing diverse views across multiple Muslim communities, having a majority of female representatives, which given higher tobacco use among men could have weakened the validity of the key points raised, and not reporting on demographic characteristics for public involvement members. Furthermore, few explicit views about non-cigarette tobacco use such as smokeless tobacco and Shisha were provided by representatives, which, while common in minority ethnic groups, is not likely to be adequately addressed by existing National Health Service (NHS) stop smoking support. Future investigations should include qualitative work to verify and expand on these findings.

## Conclusion

There is a need for further research into culturally acceptable tobacco cessation interventions for Muslims in the United Kingdom and beyond, including exploring the feasibility of a Ramadan-oriented tobacco cessation intervention. Given uncertainty around whether Ramadan is a good time to initiate a quit attempt or not, providing support beforehand, in preparation for a tobacco-free Ramadan, or capitalizing on progress made in reducing tobacco during Ramadan to support a quit attempt afterward, may be effective approaches and would expand the range of tailored tobacco cessation support options. Ultimately, the diversity of Muslim communities reinforces the importance of systematic co-development and piloting to maximize impact.

## Data Availability

As this was a public involvement project, data are not available.
